# Application and utilization of fermentation as a processing tool to mitigate protein putrefaction in plant-based diets

**DOI:** 10.3389/fmicb.2025.1638378

**Published:** 2025-08-01

**Authors:** Ihsaan Panapparambil Sooraj, John Leech, Tom F. O’Callaghan, Olivia McAuliffe

**Affiliations:** ^1^Teagasc Food Research Centre, Moorepark, Fermoy, Ireland; ^2^School of Food and Nutritional Sciences, University College Cork, Cork, Ireland; ^3^School of Biological Sciences, Queen’s University Belfast, Belfast, Northern Ireland; ^4^Teagasc Climate Centre, Moorepark, Fermoy, Ireland

**Keywords:** putrefaction, plant protein, fermentation, digestibility, nutrition

## Abstract

There is an evolving interest in the adoption and incorporation of plant proteins in Western diets as sustainable alternatives to meat consumption. This is typically motivated by the environmental and public health concerns posed by animal-based diets. However, plant proteins have been demonstrated to exhibit reduced bioavailability as a consequence of high concentrations of anti-nutritional factors (ANFs) and complex protein structures, leading to incompletely digested protein reaching the colon. This undigested protein undergoes microbial putrefaction, generating metabolites like ammonia, phenols, and hydrogen sulfide that are potentially associated with inflammation, gut barrier dysfunction, and chronic diseases. Literature searches utilizing tools such as Google Scholar and PubMed were performed with identifying relevant work in both putrefaction and fermentation, to highlight gaps for future research. There is evidence that including a microbial fermentation step in the processing of plant proteins can degrade ANFs, hydrolyze protein structure, and increase free amino acids, thereby improving upper gastrointestinal digestibility. The application of fermentation strategies can address both nutritional and safety challenges by pre-digesting proteins and enriching functional metabolites such as SCFAs and polyphenols. However, gaps persist in understanding many elements of fermentation of plant proteins including microbial consortia optimization, *in vivo* impacts, and long-term health outcomes. This review examines protein putrefaction in the gut and its association with adverse health impacts, and furthermore, fermentation is evaluated as a potential processing aid for plant proteins to enhance digestibility and mitigate putrefaction risks.

## 1 Introduction

Food production systems and consumption patterns can have significant consequences for population health, climate, and the environment. Climate change, food insecurity, and an increase in the prevalence of diseases can be associated with unhealthy dietary patterns worldwide, as well as unsustainable food systems (Willett et al., 2019). Current food production systems and agrifood practices have been identified as a major contributor to greenhouse gas emissions and biodiversity loss (Carey et al., 2023). Global meat consumption has risen significantly over the past decade, driven by population growth and increasing incomes (Godfray et al., 2018). Meat is a nutritionally dense food, providing all essential amino acids, high-quality protein, and micronutrients such as zinc, haem-iron, and vitamin B12 (Pereira and Vicente, 2013). However, livestock production systems have been associated with substantial environmental burdens, including greenhouse gas emissions, arable land use, water consumption, and soil degradation (Godfray et al., 2018). Additionally, despite its nutritional benefits, high consumption of red meat has been linked to adverse health outcomes, including cardiovascular diseases (CVDs), colorectal cancer (CRC), and metabolic disorders (MDs) (Pereira and Vicente, 2013). A global modeling study by Springmann et al. (2018) across 150 countries found that reducing animal-product consumption and moving to more sustainable diets in high- and middle-income countries could decrease mortality rates, mitigate environmental impacts from water and land use, and improve public health outcomes. On the other hand, there is some evidence from other database studies that indicates that a drastic shift to environmentally sustainable diets can cause deficiencies in micronutrients, which can especially cause issues for vulnerable members of a population like pregnant individuals and infants (Leonard et al., 2024; Leonard et al., 2023).

**Figure 1:**
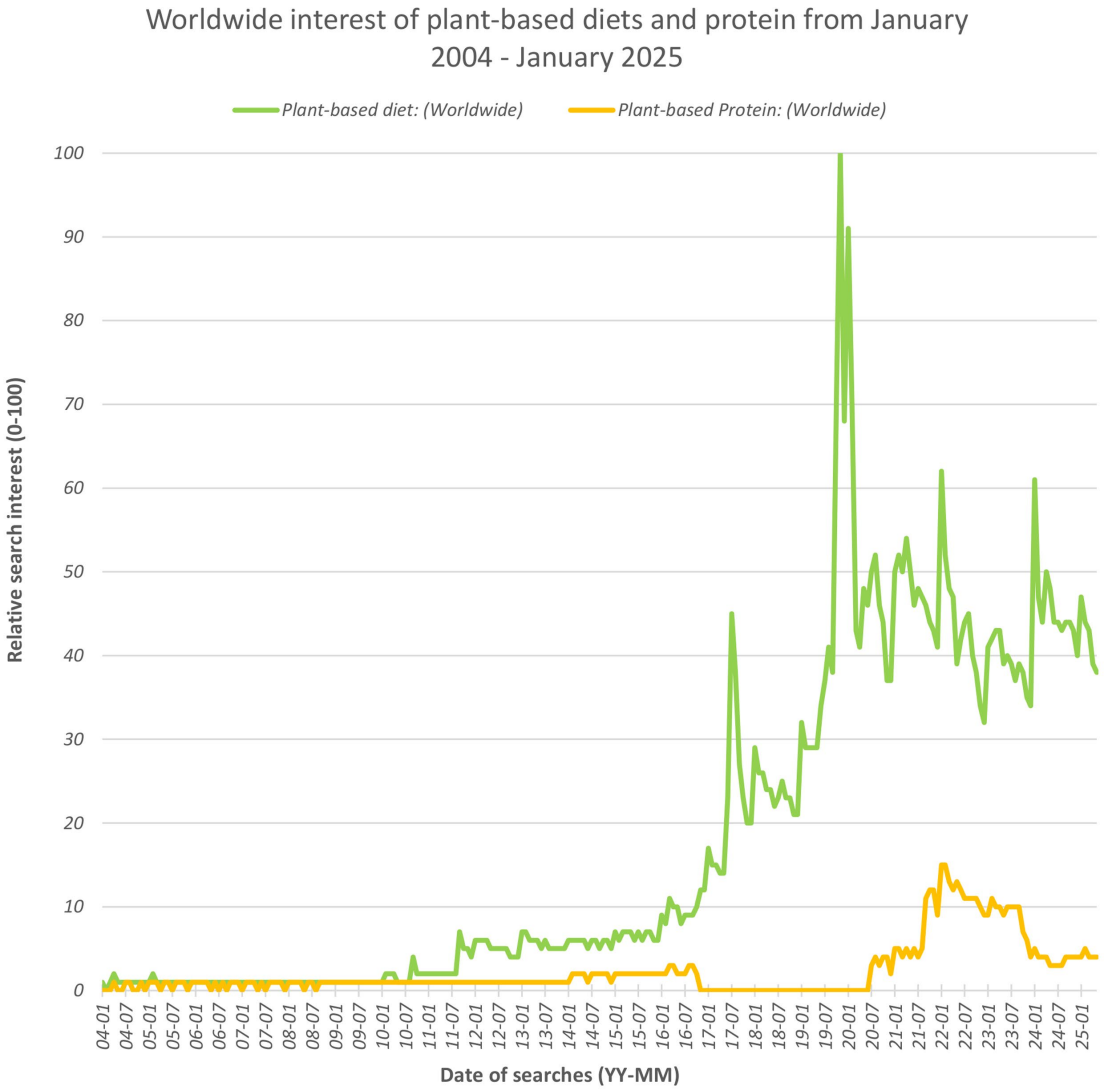
Line graph depicting the relative worldwide interest of plant-based diets and plant-based protein based on volume of searches. This data was publicly available and accessed from Google Trends (Google, 2025) and the terms used are considered by Google to be ‘topics’, equivalent terms in foreign languages are also considered within this graph.

Plant-based diets have gained momentum worldwide in the last decade, driven by health, environmental, and ethical considerations (Graça et al., 2015). Figure 1 uses data from Google Trends to illustrate the peak in public interest in plant-based diets and to a lesser extent plant-based protein, before a subsequent dip and slow down (Google, 2025). Consumer surveys in European countries showed a resistance in traditionally omnivorous individuals toward adopting more plant-based foods, with major barriers being nutrition and sensory concerns (Perez-Cueto et al., 2022). The United Nations established the 17 Sustainable Development Goals, for which UN member states have targets of reducing greenhouse gas emissions by 43% by 2030 and achieving net zero by 2050 (Kraak and Aschemann-Witzel, 2024).

Plant protein sources such as legumes, nuts, and seeds contain adequate levels of protein, but are limited by certain intrinsic factors such as cell structure and anti-nutritional factors that impact the nutritional value (Bessada et al., 2019). Plant proteins in their native form typically have reduced nutrient bioavailability than animal-based protein, demonstrating reduced anabolic muscle development, lower levels of essential amino acids leucine, lysine, and methionine, and poorer digestibility due to their protein structure compared to animal-sourced proteins like eggs, dairy, and meat (van Vliet et al., 2015). Most legumes are limited in the amino acid methionine, while grain-derived proteins are limited in lysine. Soy is an exception, as it does contain all the amino acids, however its methionine content may not be adequate for all diets (Gorissen et al., 2018; Shrestha et al., 2023). Contributing to the lower bioavailability are complex plant cell structures and the presence of anti-nutritional factors (ANFs) like trypsin inhibitors, chymotrypsin inhibitors, tannins, and phytic acid (Salim et al., 2023) that can negatively affect the processing and absorption of essential nutrients, including proteins. Plant-based proteins contain a higher proportion of β-sheet and lower α-helix structures compared to animal proteins. α-helices are more water soluble and have accessible structures, meaning they can be broken down by digestive enzymes more easily. The ANF class of trypsin inhibitors in plant material can further reduce plant protein breakdown in the upper gastrointestinal (GI) tract (Bessada et al., 2019).

Proceeding initial digestion in the stomach and small intestine, digested material enters the large intestine containing a very diverse consortia of microbial life. The gut microbiome has been documented as an essential component of human health (Piccioni et al., 2022), so much so that it has been proposed to be considered as a virtual endocrine organ affecting the function of distal organs and systems (Clarke et al., 2014; O'Hara and Shanahan, 2006). While there have been many studies and reviews focused on the role of the microbiome on saccharolytic degradation of resistant starch (RS) or fibers in the colon resulting in the generation of health-promoting metabolites (Adam et al., 2016), the degradation of undigested proteins or resistant protein (RP) by the colon microbiota, known as putrefaction, has received less attention (Wu et al., 2022). Putrefaction can lead to the production of harmful metabolites associated with a range of adverse health effects, including inflammation, intestinal barrier dysfunction, and an increased risk of chronic diseases such as colorectal cancer (CRC) and ulcerative colitis (UC) (Diether and Willing, 2019; Windey et al., 2012). In a traditional balanced diet, putrefaction is usually not an issue as meat, fish, poultry, dairy, and plant proteins diversify daily protein intake with regards to digestibility, secondary nutrients, and amino acids (Yao et al., 2016; Cummings and Macfarlane, 1991), although excessive levels of red meat consumption have been associated with an increase in putrefaction metabolites (Bingham et al., 1996). However, to meet protein demands in a plant-based diet with no animal products, more undigested plant protein would pass through the digestive system than in a balanced diverse diet. There is some evidence to suggest that plant-based proteins in particular are prone to putrefaction, creating issues for the sustainable, widespread adoption of diets containing less meat (An et al., 2014). Indeed, some studies have shown increased populations of putrefying bacteria in the colons of CRC patients compared to healthy adults, which indicates a potential correlation between inflammatory conditions and putrefaction (Wang et al., 2011). However, there is a lack of information on the *in vivo* mechanisms of putrefaction. This makes it difficult to see a direct association between gut dysbiosis resulting from the accumulation of putrefactive microbes, and human health. Furthermore, identifying mitigation strategies based on clear evidence is challenging.

There is a burgeoning interest in utilizing microbial fermentation as a method in food formulation to improve the digestibility of plant proteins. Fermentation as a food processing tool has been used for centuries across various traditions as a form of preservation. For animal products, especially dairy products such as cheese, yoghurt, and kefir, there have been many studies demonstrating the proteolytic capabilities of fermentation and subsequent improvement in digestibility (Lucia et al., 2014; Fox and McSweeney, 1996; Ferreira et al., 2010). Proteolytic enzyme treatments have also been proposed as a solution to improve digestibility of proteins, as well as host supplementation with probiotics (Goodarzi Boroojeni et al., 2017; Cerdá-Bernad et al., 2022). These proven properties of fermentation could thereby reduce the negative effects of putrefaction in the lower gut. The digestibility of plant protein was shown to have increased *in vitro* as a result of pre-processing of the plant-based protein via fermentation, hypothesized to be due to enzymatic degradation of ANFs, proteolytic activity, and modification of protein structure, which would all allow proteins to be hydrolyzed to a greater extent in the upper gut (Zhao et al., 2019; Diether and Willing, 2019; Peled and Livney, 2021).

**Figure 2:**
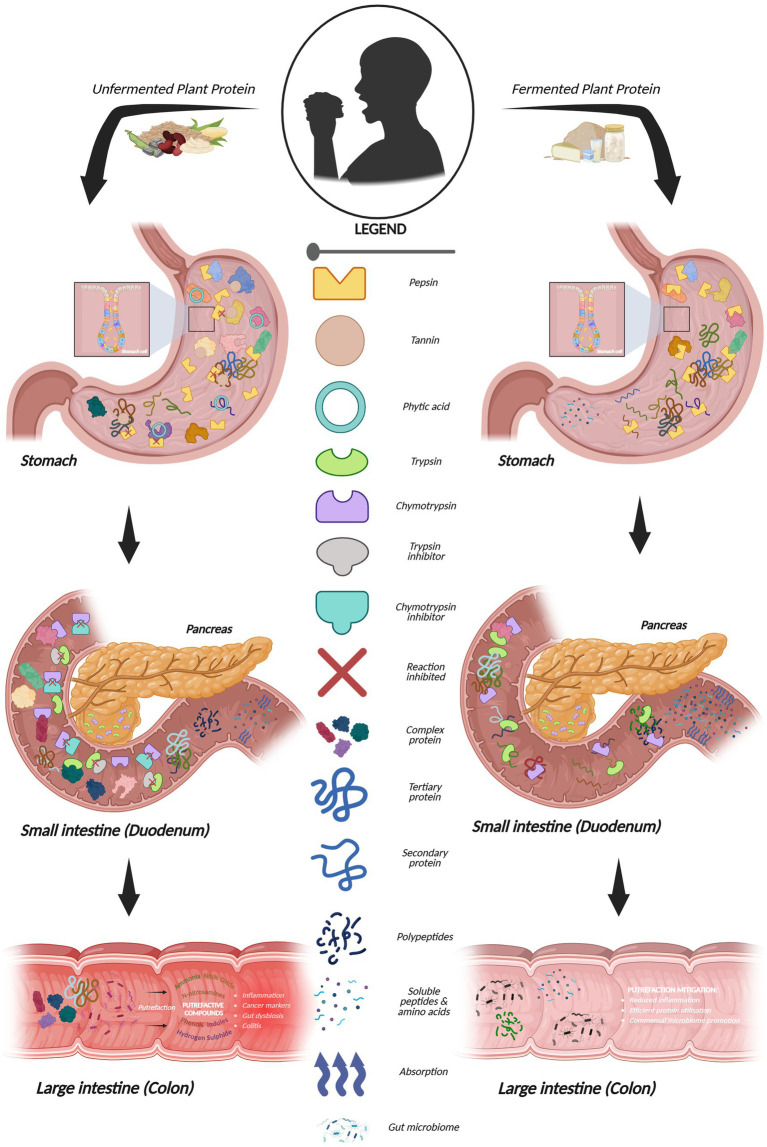
Enzymatic and microbial digestion of unfermented versus fermented protein in the human gastrointestinal tract depicted in infographic form. Larger degree of undigested protein in unfermented protein remains due to inhibition of digestive enzyme activity leading to increased putrefaction in the colon compared to fermented protein. This can lead to many different disease factors as depicted in the colon. Created in BioRender. Panapparambil Sooraj, I. (2025) https://BioRender.com/dvuz4mi.

This narrative review examines the potential of fermentation strategies as a processing tool to enhance the nutritional qualities of plant proteins, in turn alleviating some of the negative effects associated with putrefaction. Specifically, it will examine the mechanisms by which putrefaction produces toxic compounds having a subsequent effect on human health, how fermentation modifies plant protein structures and enhances digestibility, and highlights gaps in the current knowledge, identifying pertinent future research directions for the field. The review presents a potential solution in fermentation (see Figure 2) to the less-understood issue of protein putrefaction in the colon, the current state of research of both, and how they might interlink, and future perspectives on understanding the specific mechanisms of their interactions.

## 2 Methods

While this review is narrative, seeking to highlight a potential gap in knowledge in the field of plant-based nutrition, systematic review approaches have been utilized. Searches were conducted to identify research from two broad areas: (1) protein putrefaction, or (2) colonic fermentation of protein and fermentation of plant protein. The aim of presenting the searches as they were used exactly, is to improve reproducibility of this methodology as this burgeoning field progresses.

### 2.1 Putrefaction

A literature search was performed in the Medline PubMed database and Google Scholar to assess current research on protein putrefaction, effects on gut microbiota, and overall health. The specific search terms were used, along with excluding non-English papers and pre-prints:

((Protein[Title]) AND (Putrefaction[Title] OR fermentation[Title] OR decomposition[Title])) AND ((Gut[Title/Abstract] OR Colon[Title/Abstract] OR Intestine[Title/Abstract]) OR (Health[Title/Abstract] OR Nutrition[Title/Abstract] OR Disease[Title/Abstract]) OR (Microbiome[Title/Abstract] OR Microbes[Title/Abstract] OR Microbiota[Title/Abstract] OR Flora[Title/Abstract]))

Google Scholar has an “advanced search” feature which was utilized instead of Boolean operators. The final search query was:

protein gut putrefaction microbiome fermentation “protein putrefaction.”

Papers were shortlisted from 150 results from PubMed and 120 results from Google Scholar, based on relevance to protein putrefaction or fermentation in the colon, and/or fermented plant material. The selected studies mainly include *in vitro* experiments; utilizing human-sourced colon microbes, and *in vivo* animal studies, primarily in rats and mice, due to their widespread use as model organisms, and pigs as they are a promising model of digestive disease (Gonzalez et al., 2015). The remaining studies include clinical trials examining the role of putrefaction in IBD patients, and molecular studies examining the interactions of protein putrefaction products with intestinal epithelial cells (utilizing models like human Caco-2 cells, and rat intestinal epithelial cells). Review articles were used to understand the current depth of the field, and further references were obtained from those bibliographies.

### 2.2 Fermentation of plant-based protein

A specific literature search was performed in the Medline PubMed database and Google Scholar to identify papers involving fermentation of plant material and the effect on digestibility of protein. Non-English-language papers, and pre-prints were excluded from the search. The search query utilized was:

((Plant[Title/Abstract] OR Bean[Title/Abstract] OR Grain[Title/Abstract] OR Legume[Title/Abstract] OR Vegetable[Title/Abstract] OR Pea[Title/Abstract]) AND (Protein[Title/Abstract])) AND (Fermentation[Title]) AND (Digestibility) NOT (Silage[Title/Abstract]) NOT (Rumen[Title]) NOT (Ruminant[Title]) NOT (Meat[Title]) NOT (Ruminal[Title])

Google Scholar “advanced search” was used instead of Boolean operators, and the search query was:

allintitle: protein fermentation digestibility plant OR legume OR bean OR grain OR vegetable OR soy OR faba OR rice OR dough OR wheat OR barley OR rye OR pea OR ANF OR antinutritional OR nutrition -silage -rumen -ruminal -meat -ruminant.

Papers were shortlisted from 98 results from PubMed and 54 from Google Scholar, based on their applicability toward human food as many papers were focused on animal feed. The selected studies mainly include *in vitro* digestibility experiments on foods processed by fermentation, analysing digestibility, ANFs, and sensory characteristics. References from review articles were also utilized, including those that examined the nutritional impact of plant protein fermentation.

## 3 The production of putrefactive compounds in the gut

According to the World Health Organisation (WHO) (World Health Organisation, 2007), an average adult human should consume 10–15% of their daily calories in the form of protein, representing about 0.83 grams of protein per kilogram of body weight. Protein is an incredibly important component of the diet, needed for normal bodily function, cell repair, and growth (World Health Organisation, 2007). However, the digestibility of proteins in the upper gut or stomach can vary significantly due to a range of intrinsic and extrinsic factors including the structure or three-dimensional folding of the protein, the composition of amino acids, interaction with other components (such as ANFs), post-translational modifications, and polypeptide charges (Gorissen et al., 2018; van Vliet et al., 2015). These variations in digestibility can be a result of the source of protein, how it is processed, other dietary components, and host health.

Initial digestion of proteins occurs in the stomach and small intestine, mediated by pepsin, trypsin, and chymotrypsin. Some undigested proteins and peptides entering the large intestine are further digested by pancreatic enzymes, and the metabolic products are absorbed by the gut microbiota to provide energy and components for cell proliferation via protein production. The human gut microbiome is incredibly diverse, with taxa capable of protein putrefaction identified including *Actinomyces*, *Bacteroides*, *Clostridium*, *Fusobacterium*, *Peptostreptococcus*, and *Propionibacterium* (Kaur et al., 2017). Putrefaction can result in the release of a plethora of potentially harmful metabolites including nitrogenous compounds such as ammonia (NH_3_), *N-*nitrosamines, amines, phenols, cresols, and indoles (Yao et al., 2016; Korpela, 2018). The effects of these metabolites on health will be explored in a later section. Putrefaction can also result in products associated with positive health effects such as short-chain fatty acids (SCFAs) and branched-chain fatty acids (BCFAs) via reductive deamination, or the removal of an amino group. Certain SCFAs, such as butyrate, have been shown to be an important energy source for colonocytes. Butyrate is also implicated in the inhibition of CRC and inflammation (Musch et al., 2001). While SCFAs are also produced via the degradation of fibers and starches, BCFAs are exclusively produced from the deamination of the branched-chain amino acids valine, leucine, and isoleucine, resulting in isobutyrate, isovalerate, and 2-methylbutyrate, respectively. The role of BCFAs was poorly understood, but there is now clinical research showing their involvement in signaling, intestinal maintenance in neonates, and general microbiome health (Lu et al., 2024).

## 4 Toxicity of putrefaction products and their impact on human health

Due to the limitations of studying real-time gut activity, human studies are sparse. However, animal studies, particularly those using pigs as a model of human gastrointestinal activities, are valuable. These studies often look at concentrations of specific toxic compounds remaining in the feces, urine, or present in the gut. The concentration of these compounds often determines their toxicity to the host organisms, which is inversely related to the ability of the colon to absorb and detoxify the compounds. There are potential thresholds below which these compounds would not necessarily be deleterious to health in otherwise healthy subjects, as they would be inactivated by the colon. This is based on the understanding of colon and liver function, depending on the on their ability to detoxify these compounds, which can vary from individual to individual (Yao et al., 2016). However, there is a lack of clarity on the effective toxicity of these compounds in humans, current understanding is based on animal and *in vivo* studies and these thresholds are not well established (Windey et al., 2012).

### 4.1 Nitrogen compounds

Ammonia (NH_3_) released in the human gut is a by-product of bacterial deamination of amino acids. Besides being a product of putrefaction, endogenous nitrogen recycling results in constant exposure of epithelium cells to NH_3_. As reported in the literature, increased concentrations of fecal ammonia have been associated with increased consumption of crude protein (Yao et al., 2016). Patients with CRC also present with increased concentrations of fecal ammonia (Kaur et al., 2017). Caco-2 cells incubated with ammonia showed an increase in cell proliferation and increase in epithelial permeability (Hughes et al., 2008). This increase in permeability can cause mutations in RNA, indicating potential mutagenic and carcinogenic properties of NH_3_ proportional to its concentration (Hughes et al., 2008; Hughes et al., 2000). In rat model studies, elevated ammonia levels resulted in reduced absorptive capacity of colonocytes, which may potentially reduce detoxification rates (Roediger and Babidge, 1997). Increased ammonia production in piglets was seen after a 2.5X increase in the *Clostridium leptum* group in the gut compared to the control. This was due to feeding piglets high amounts of crude protein (Levesque et al., 2014). These studies indicate that excess ammonia demonstrates some negative effects on gut health, but not a specific connection between ammonia and gut disease like CRC. Another study involving human biopsies from the colon showed that toxic effects of NH_3_ may potentially be counteracted by butyrate (Bartram et al., 1993). Excess NH_3_ can enter the bloodstream through epithelial capillaries and be detoxified via the urea cycle in the liver and be excreted in urine (Walker, 2009). However, if detoxification via liver or colon does not match up to production, an accumulation of harmful metabolites is possible.

Nitric oxide (NO) can be produced both endogenously and via intestinal microbiota in the colonic mucosa (Sobko et al., 2005). Endogenously produced NO uses arginine as a precursor and is used in signaling in the gut for motility and production of mucus. Increased levels of NO are associated with IBD and UC, potentially due to increased microbiota activity in individuals with these conditions. However, small doses of NO have been shown to improve ionic movements in pig epithelia, as well as preventing cellular infiltration in chemically induced colitis in rats (Armand et al., 2022; Jha and Leterme, 2012).

Decarboxylation of amino acids by microbes produces amines in the gut, which can then be further metabolized by the microbiota leading to formation of N-nitrosamines (Sobko et al., 2005; Yao et al., 2016). Species of *Lactobacillus*, *Streptococcus*, *Bacteroides*, and *Clostridia* found in the gut can ferment arginine into ornithine. Ornithine is then converted to the polyamines including putrescine, spermidine, and spermine (Kaur et al., 2017). These compounds can be involved in mucosal proliferation in rats (McCormack and Johnson, 1991), and small intestinal absorption in piglets (Tan et al., 2024). Putrescine was found to be expressed in proximal regions to intestinal obstruction in rat colons, causing a hyperplastic change (Kaur et al., 2017; Seidel et al., 1984). It was also shown to be involved in downregulation of a butyrate transporter in piglets, leading to potential energy losses in colonocytes. The endogenous conversion of amines to *N*-nitrosamines is associated with increased fecal water genotoxicity and colonocyte DNA mutations, which are signs of increased CRC risk. The main risks from these compounds appear to be DNA damage, which has only been examined in livestock (Andriamihaja et al., 2015). As these animals are often slaughtered young, there is no data to indicate whether tumorigenesis occurs down the line.

### 4.2 Phenols and indoles

Phenols and indoles are produced in the colon via the degradation of aromatic amino acids tyrosine, phenylalanine, and tryptophan (Pedersen et al., 2002; Armand et al., 2022). These compounds are absorbed and detoxified by mucosa in the colonic lumen, within the cytosol, where they undergo sulphation driven by sulphotransferases (Armand et al., 2022; Roediger and Babidge, 1997). One of the toxic compounds from tyrosine, *p-*cresol, is excreted in the urine and has been shown to be a reliable indicator of the degree of degradation of aromatic amino acids. 90% of all phenolic compounds are excreted in the urine as *p*-cresol (Andriamihaja et al., 2015). An increase in luminal phenol has been linked to high-protein diets in rats, pigs, and humans as determined by *p-*cresol concentrations in feces and urine. *In vitro* cultures of Caco-2 cells incubated with phenols showed a rapid increase in tight junction (TJ) permeability, tested with mannitol transfer across epithelial cells. This increase is associated with tumor formation due to the role of cell permeability in signaling. This increase was proportional to dosage of phenols, as well as time in contact. 90% of phenolic compounds are broken down into *p-*cresol and expressed in urine and feces (Hughes et al., 2000). In one study, caecal concentrations of putrefactive compounds in rats were measured after feeding with casein, fish meal, and soy protein. Soy protein showed the highest degree of putrefactive compounds, with casein showing the least putrefaction (An et al., 2014). Another study in rats showed that DNA damage of colonocytes was positively associated with an increase in *p-*cresol and phenol concentration in stool, which could be alleviated by introduction of RS into the diet (Toden et al., 2006). Phenol toxicity was seen in *in vitro* colonic epithelial cells treated with phenols, as well as in patients with UC, demonstrating a potential link to protein degradation products and the disease (Nemoto et al., 2012).

Phenolic and indolic compounds are processed by absorption and conjugated both in the mucosa of the colonic lumen, or in the liver via epithelial capillaries (Windey et al., 2012). This is part of the body’s “detoxification” process, but there is some evidence that the conjugated *p-*cresyl sulfate and indoxyl sulfate are involved in vascular and renal disease progression. The unconjugated indoles may have beneficial effects on the colon, having an opposite effect on tight junctions compared to phenols. This was demonstrated in germ-free mice, where indoles acting as quorum-sensing molecules promoted expression of junction-related mRNA in the mucosal lining, thus strengthening it (Armand et al., 2022; Shimada et al., 2013).

Phenols are fermentation products of tryptophan, which is also fermented in the gut to produce serotonin. Serotonin’s primary role is as a neurotransmitter, and it is thought to play an important role in the gut–brain axis. There have been some studies showing potential links to protein putrefaction and neurological conditions like schizophrenia (Liang et al., 2022; Sanctuary et al., 2018). Serotonin increase in healthy humans was associated with strengthened mucosal barriers. This increase was not seen in patients with IBD, indicating a potential dysfunction in the signaling pathway in diseased patients (Ghia et al., 2009). These compounds are yet to be directly associated with any diseases but have been demonstrated to play a signaling role in epithelial function of the colonic mucosa. However, urinary and fecal *p-*cresol show potential in use as a screening tool for inflammatory conditions or protein putrefaction (Andriamihaja et al., 2015; Yao et al., 2016).

### 4.3 Hydrogen sulfide

Other gaseous by-products of protein fermentation include hydrogen (H_2_), methane (CH_4_), carbon dioxide (CO_2_), and hydrogen sulfide (H_2_S). Of these, H_2_S is considered very toxic, produced by fermentation of sulfate and sulfur amino acids like methionine, cysteine, cystine (dimer of cysteine) and taurine (Hughes et al., 2000; Yao et al., 2016). Among the metabolites of distal colonic fermentation, H_2_S is associated with the most markers for inflammatory conditions. Sulfide concentrations in urine and feces are positively correlated with increased protein intake in humans, and in UC patients versus healthy patients. Reduction of foods with sulfates and sulfur-containing amino acids showed improvement in UC symptoms, in a human pilot study (Yao et al., 2016). Recently, another study was conducted where patients consumed low-sulfur diets and received fecal microbial transplants. This showed a significant decrease in symptoms, more than either treatment on its own. Using a human carcinoma epithelial model, sodium hydrogen sulfide was found to inhibit cellular oxygen consumption, proliferation rate, and uncoupling of mitochondrial respiration. While this affected ATP usage of epithelial cells, they were still viable, potentially due to adaptive responses.

H_2_S has also been implicated in driving CRC. Increased apoptosis and proliferation of the epithelial layer was observed in human rectal biopsies, and rat small intestinal cells following incubation in the presence of H_2_S (Linden, 2014).

## 5 Microbes responsible for putrefaction in the gut

As previously described, the composition and diversity of the human gut microbiome influence the degree to which protein putrefaction occurs. In addition, consumption of different types of protein and their level of processing prior to consumption, whether by heat treatment, fermentation, or enzymatic processes, can influence this composition and diversity, with different gut bacteria of varying levels of pathogenicity (Yao et al., 2016). *In silico* analysis of bacteria based on the putrefaction pathways of all major colonic fermentation products demonstrated that the genus *Fusobacterium* was abundant in most of the CRC-related datasets compared to healthy adult datasets (Kaur et al., 2017). It was identified as being a risk factor for carcinoma progression in the colon and contains genes coding for a high number of putrefaction pathways associated with toxicity. While all analyzed pathogenic bacteria were found to have some putrefaction pathways, *Streptococcus, Candidatus, Escherichia, Shigella, Prevotella*, and *Selenomonas* were found to be in particularly higher levels in patients with CRC. Some commensal gut bacteria were also seen to have putrefaction pathways. Putrefaction by those bacteria may contribute to positive gut health with low levels of putrescine or indole as discussed previously (Kaur et al., 2017).

## 6 Prevalence of putrefaction on different protein types

High levels of animal protein in a diet are associated with a reduction in saccharolytic members of the phylum *Firmicutes*. These microbes are normally associated with SCFA and BCFA production, reducing inflammatory factors in the gut. The lack of propionate and butyrate leads to a favorable environment for growth of pathogenic bacteria, including *Escherichia* and *Enterococcus* (Stecher and Hardt, 2011). The increase in non-digestible oligosaccharides in the form of soy meal in weaning piglets led to an increase in *Bifidobacterium* (Le Leu et al., 2007). Soybean is a source of complete proteins; it contains all essential amino acids. When fed to rats, it also demonstrated a potential prebiotic effect with increases in lactobacilli and bifidobacteria with a decrease in pathogenic bacteria (Bai et al., 2016). Despite these seemingly positive signs, the higher bioavailability of animal protein such as casein or white meat seems to lead to higher digestibility and less putrefactive metabolite production than soy protein. A lower abundance of *Fusobacteria* was found in rats fed casein, egg, and white meat protein as compared to plant protein, and both dairy and meat protein fed to rats at recommended levels promoted growth of members of *Lactobacillaceae,* and *Bifidobacterium*, compared to soy at similar levels (An et al., 2014). Zhu et al. (2025) compared *in vitro* fermentation of sheep whey protein versus soy protein isolate using fecal microbiome from healthy volunteers. The authors suggest that the increase in acetic acid in sheep whey protein is due to potential commensal strains that enter the glycolytic pathway. Sheep whey protein also showed higher SCFA, butyric, isobutyric, and isovaleric acids compared to soy protein isolate (Zhu et al., 2025). Despite the presence of RS, the high level of undigested protein in a meal primarily consisting of soy protein leads to putrefaction. Soybeans also contain a large amount of trypsin inhibitors, ranging from 16 to 27 mg/g in raw soybeans (Vagadia et al., 2017), so without removal or reduction of ANFs, this can lead to poor digestion and absorption.

## 7 Protein processing tools to improve digestibility

Protein structures and chemical composition can be modified by different processing tools, whether to make protein less or more easily digestible. Non-thermal methods like germination (Jawanda and Ramaswamy, 2024), sonication, pulsed electric field, and high pressure can potentially reduce prevalence of ANFs and cause protein structures to unfold, improving digestibility (Kamiloglu et al., 2024). Similarly, thermal processes like cooking, extrusion, autoclaving, drying, and freezing can break down ANFs and unfold complex protein structures (Skalickova et al., 2022; Purwandari et al., 2024).

Plant protein digestibility can be increased through these mild non-thermal methods that unfold the protein, exposing hydrolysis sites and reducing ANFs. There is an option of utilizing these tools in tandem with fermentation to drive further protein processing (Goodarzi Boroojeni et al., 2017; Jawanda and Ramaswamy, 2024), or allowing access to reaction sites otherwise unreachable by microbes. There are a wide variety of plant protein sources with distinct protein and chemical structures that can be affected by these methods in a variety of ways, so it is important to be specific and consider the fact that these independent proteins are often not the only factor in a diet which can affect digestibility.

## 8 Fermentation as a processing method for plant proteins

As described previously, fermentation is a traditional food processing technology predating domesticated agriculture. Traditionally, it was a form of extending the shelf life of foods prior to the widespread availability of cold storage (Ross et al., 2002). Now, while it is still utilized for preservation, it is also employed as a biotransformation tool to improve sensory characteristics and nutritional value, with the latter being a topic of interest in recent research. Some plant fermentations like the fermented cabbage kimchi (Kim et al., 2023), fermented whole soybean cake tempeh (Ahnan-Winarno et al., 2021), or fermented tea kombucha (Değirmencioğlu et al., 2021) all demonstrate added value through improved antioxidant content, digestibility, and bioaccessibility. However, there has only been recent renewed research interest in plant protein fermentation, with the intention of creating a highly digestible fermented product with comparable protein quality to animal protein. Heat-labile ANFs like trypsin inhibitors and lectins (as discussed previously) are readily reduced through high thermal or extrusion cooking in conventional plant protein processing. However, heat-stable ANFs, like phytic acids and phenolic compounds, remain in the product, worsening digestibility and potentially leading to putrefaction. Some studies involving fermentation of plant protein will be explored, along with their effects on overall nutrition.

As such, Table 1 depicts a variety of examples of experimental studies exploring the impact of fermentation of plant protein directly on protein digestibility. The limitations of some of this data are that there is a lack of replication of their work, as well as a lack of controls or sterilization of substrates used that could confound collective conclusions. Furthermore, many of these studies also look at ‘natural’ or spontaneous fermentations, which are challenging to experimentally replicate.

**Table 1:** Examples of fermentations of plant-based protein in literature and observed effects on protein digestibility.

Plant substrate	Organism	Fermentation types	Effects on protein digestibility	References
Baobab seed African locust bean	Spontaneous fermentation	SSF	*In vitro* protein digestibility of baobab seed increased slightly by ~5% and significantly in locust bean by ~30%	Addy et al. (1995)
Black mash bean	*S. cerevisiae* *Lactobacillus* spp.	SSF	*In vitro* protein digestion increased with prolonged fermentation time; *Lactobacillus* showed higher digestibility	Ali et al. (2024)
Cassava leaf Babassu palm mesocarp	*R. microspores* var. *oligosporus*	SSF	PDCAAS of a fermented mixture containing 32% cassava leaf and rest babassu mesocarp showed results that were comparable to legume-based proteins	Morales et al. (2018)
Chickpeas	*Lpb. plantarum*	SMF	Chickpea macromolecule protein demonstrated to be hydrolyzed during fermentation. Looser states of structures would enable better digestibility	Liu et al. (2023)
Chickpeas	*P. pentosaceus*	SSF	Significant decrease in ANF phytic acid, *in vitro* digestibility increased to >80% from ~74% in both strains	Zhou et al. (2025)
Chickpeas	*B. subtilis*	SSF	Chickpea proteins were degraded to small peptides, and a release of soluble proteins and proteinase activity was seen	Li and Wang (2021)
Fava bean	*Bacillus pumilus*	SSF	ANF concentrations were significantly decreased, amino acid, and soluble protein concentrations were significantly increased	Xu et al. (2023)
Finger millet	Spontaneous fermentation	SMF	*In vitro* protein digestibility increased by 23%, ANFs significantly reduced	Usha and Chandra (1998)
Fluted pumpkin seed	Spontaneous fermentation	SSF	*In vitro* protein digestibility increased by ~20% by day 5 but decreased by ~10% from that point by day 7. This corresponds to protein efficiency ratio when fed to rats	Giami (2004)
Foxtail millet	*Lpb. plantarum*	SMF	Protein breakdown observed, increased protease activity, slight increase in soluble protein content, with 12 and 14% higher *in vitro* gastric and intestinal digestion	Bai et al. (2025)
Kidney bean, black bean Oat	Oyster mushroom (*Pleurotus ostreatus*)	SSF	*In vitro protein digestibility* was increased—39.99–48.13% in black beans, 44.06–69.01% in kidney beans, and 63.25–70.01% in oats ANFs were reduced, and amino acid content was improved	Espinosa-Páez et al. (2017)
Lentils Chickpeas Fava beans	*A. oryzae* *A. niger* *Lpb. plantarum*	SSF	While hydrolysis was observed to have occurred, digestibility had decreased across all fermentations. Potentially due to presence of natural microbiota	Stone et al. (2024)
Lentils Quinoa	*Oyster mushroom (P. ostreatus)*	SSF	*In vitro* protein hydrolysis of sample increases up to 20% Small peptide proportion after *in vitro* digestion increased up to 35%	Sánchez-García et al. (2024)
Lupin	*P. pentosaceus*	SSF	*In vitro* protein digestibility was higher in both fermentation types, reduced TIA activity was also observed	Bartkiene et al. (2018)
Moth bean	Spontaneous fermentation	SMF	ANFs phytic acid, polyphenols were decreased Protein digestibility increased at both temperatures up to 80% from 60%	Bhandal (2008)
Pea	*Lpb. plantarum* *Llb. brevis*	SMF	Protein breakdown observed, PDCAAS improved while overall protein content remained similar as unfermented	Kim et al. (2024)
Peas	*Lpb. plantarum*	SMF	*In vitro* digestibility was shown to have increased significantly after fermentation	Skalickova et al. (2022)
Roselle/Karkade	Spontaneous fermentation	SSF	*In vitro* protein digestibility increased by ~15% after 6 days, then decreased after 9 days	Yagoub et al. (2004)
Sicklepod	Spontaneous fermentation	SSF	One of the two samples tested (Algeninan) had protein digestibility increase to 50%, while the other (Zalngy) decreased to 30%	Osman et al. (2010)
Sorghum	Assorted LAB	SMF	*In vitro* protein digestibility was shown to be higher by ~5% for samples fermented with LAB consortium compared to that fermented spontaneously	Ogodo et al. (2019)
Soybean	*Lsl. fermentum* *Pichia fermentans*	SMF	Large peptides were degraded, with increase in FAAs	Cao et al. (2025)
Soybean	*A. oryzae* *B. subtilis*	SSF	*In vitro* digestion of both samples increased, with fermentation by *B. subtilis* increasing it further	Teng et al. (2012)
Soybean	*Bacillus siamensis*	SSF	Trypsin inhibitor amounts significantly decreased, *in vitro* digestibility and absorbability increased slightly, while soluble protein increased significantly	Zheng et al. (2017)
Soybean	*Bacillus amyloliquefaciens*	SSF	*In vitro* digestibility increased by ~10% in samples fermented with this isolated strain, and increased acid soluble protein content	Li et al. (2020)
Soybean	*B. subtilis* subsp*. natto*	SSF	*In vitro* gastrointestinal digestion model showed increase acid soluble peptide yield – indicating higher digestibility. Large proteins were broken down into smaller structures as well	Ketnawa and Ogawa (2019)
Victoria beans	Spontaneous fermentation	SMF + SSF	Total protein content decreased in all fermentations with an increase in *in vitro* digestibility from a range of 4–8%	Granito et al. (2002)

SSF, solid state fermentation; SMF, submerged fermentation.

### 8.1 Fungi

Aspartic proteases produced by filamentous fungi and some yeasts are specifically active against aromatic amino acid residues, again seen primarily in cheese making from *Mucor* spp. in ripening. “Pepsin-like” or “rennin-like” enzymes produced from other fungal genera like *Aspergillus* or *Rhizopus,* called aspartyl proteinases, have been isolated and purified from these strains and utilized for a variety of applications (Horimoto et al., 2009). Neutral proteases from *Rhizopus arrhizus* have been used in debittering cheeses (Lucia et al., 2014).

Soybean residue, okara, is a by-product of tofu and soymilk production, traditionally used to prepare an Indonesian fermented product, oncom. It was fermented by the yeasts *Kluyveromyces marxianus* and *Cyberlindnera saturnus* in two separate studies. During these fermentations, it was generally observed that “beany” odors were reduced or removed as a consequence of ANF degradation (Christensen et al., 2022). A combined SSF of okara conducted with *Rhizopus microsporus* var. *oligosporus* and *Yarrowia lipolytica* showed a significantly higher increase in free amino acids compared to either strain monoculture, indicating a synergistic effect (Vong et al., 2018).

Solid-state fermentation (SSF) of soaked, dehulled, and boiled red kidney beans with *R. microsoporus* var. *oligosporus* resulted in an overall increase in protein digestibility, solubility, and free amino acids after *in vitro* intestinal digestion and *in vivo* buccal digestion compared to an unfermented control. This was hypothesized to be due to the breakdown of cotyledon cell wall integrity (Sun et al., 2023). *Canavalia ensiformis* seeds or Jack beans were fermented with *R. microsoporus* var. *oligosporus* via a traditional tempeh fermentation method. Protein digestibility was seen to have been improved *in vitro* compared to control cooked beans. Less undigested protein was found in the fermented sample compared to the control (Purwandari et al., 2024). Oilseed meals are a commonly discarded waste product in the cooking oil industry, and much work has been conducted on their valorisation. *Aspergillus niger* and *oryzae* were shown to improve nutritional value by adding microbial mass; however, this is being explored as an avenue for animal feed (Li C. et al., 2023).

### 8.2 Bacteria

Even though plant-based fermentations are long-standing technologies, there is limited information on the impact that microbial proteases have on the nutrition. Different types of microbes are associated with different extracellular microbial proteases, usually adapted to specific substrates. Cell envelope proteinase (CEP) and subtilisins are well-characterized enzymes in LAB seen prominently in the dairy industry. As a result, much of the research has been conducted on dairy strains, with casein as the focus (Lucia et al., 2014; Christensen et al., 2022). There are some commercial enzymes extracted from *Bacillus* strains like *Bacillus licheniformis* as well, which have shown promise in the hydrolysis of plant protein.

Many *Bacillus* species are QPS-approved under certain conditions, usually to be used only in inactivated forms. They are also widely used in enzyme production, some of which is used in the food industry. Despite their versatility, they are yet not utilized widely in commercial starter cultures (Hazards et al., 2025) (Li Z. et al., 2023). *Bacillus subtilis* SSF of fermented ground pea dough showed reduction of trypsin inhibitor activity, and phytic acid (Goodarzi Boroojeni et al., 2017). *Bifidobacterium longum*-fermented red quinoa drinks showed an increase in polyphenol and antioxidant capacity (Cerdá-Bernad et al., 2022). Several more examples are presented in Table 1, and while other traditional examples of bacterial fermentations exist in the literature, they do not tackle protein digestibility but rather focus on sensory characteristics and volatiles. For example, traditional bean pastes like Gochujang utilize *Bacillus* strains in starter cultures; however, studies focus on optimizing quality production for industry (Kim et al., 2010). For animal feed, a *B. licheniformis* strain was shown to increase the *in vitro* protein digestibility of a common waste product – oil flaxseed, hempseed, and pumpkin seed cakes (Rambu et al., 2024).

In a study exploring fermentation of cauliflower and bean mixture with multiple *Lactiplantibacillus plantarum* strains, a slight increase in some amino acids was seen with one strain (Thompson et al., 2020). Different fava bean doughs made from seeds from regions of Italy were fermented with *Lpb. plantarum* as a solid-state fermentation. A significant increase in free amino acids as well as improved *in vitro* protein digestibility was seen after 48 h of fermentation. No significant differences were seen in phenol and antioxidant content. Trypsin inhibitor activity was completely removed for most fermented doughs. Vicine was also shown to be degraded, indicating proteolytic activity (Verni et al., 2019). The air-classified protein fraction of fava bean flour was made into a dough and inoculated with *Lpb. plantarum*. Compared to the incubated, non-inoculated fraction, vicine and convicine were decreased by more than 91%, and trypsin inhibitor activity and condensed tannins were reduced by more than 40%. Phenol and phytic acid contents were not changed significantly. Free amino acids and *in vitro* digestibility were improved in fermented samples versus controls (Coda et al., 2015). *In vitro* digestibility of gluten-free bread developed with freeze-dried fava bean flour fermented with *Lpb. plantarum* strain increased by ~19% compared to bread made with unfermented fava dough. All essential amino acids were raised to values higher than unfermented fava bread (Sozer et al., 2019).

A co-culture of *Levilactobacillus brevis* and *Lpb. plantarum* was inoculated in a glucose-supplemented pea protein isolate mixture. The fermented product was shown to have a higher amino acid score with similar amounts of crude protein to the unfermented product (Kim et al., 2024). Beverages made from mung bean, kidney bean, and a combination of both in water were fermented with *Lacticaseibacillus casei*. At optimal fermentation conditions, a > 40% decrease in the ANFs, tannin, saponin, and phytate was seen in all beverages while maintaining some phenol concentration. Protein digestibility was increased, with identified proteolysis based on free amino acids and hydrolysis (Chaturvedi and Chakraborty, 2022).

*Pediococcus acidilactici, Pediococcus pentosaceus*, and *Latilactobacillus sakei* were used in individual SSFs with two varieties of lupin and soy doughs. All fermented lupin doughs showed an increase in *in vitro* digestibility on average of 18.3% and soy doughs 15.9%. Biogenic amines phenylethylamine, spermine, and spermidine were degraded, and putrescine, histamine, and tyramine were produced (Bartkiene et al., 2015).

Fermentation of a soy protein isolate slurry with three commercial *Lactobacillus helveticus* strains showed degradation of protein. LC–MS/MS and GC–MS analysis of the proteins showed that β-conglycinin α subunit 1, β-conglycinin α’ subunit, glycinin G1, and 2S albumin were specific to *Lb. helveticus* enzymes (Shirotani et al., 2021). Fermenting soy protein isolate with *Lb. helveticus* showed reduced immunoreactivity of soluble protein β-conglycinin by denaturation in acidic conditions, as well as potential proteolysis (Meinlschmidt et al., 2016). Furthermore, there was a decrease in volatile organic compounds, potentially associated with ANFs. Another fermentation of soy utilizing the same microbe showed proteolysis of more soluble proteins like glycinin and albumin. A breakdown of hydrolysis sites was seen, indicating potentially easier access by digestive enzymes in the gut (Shirotani et al., 2021).

Despite all of these positive signs, mainly indicating improved *in vitro* digestibility, many other LAB strains show poor proteolytic activity with legume proteins (Christensen et al., 2022). There is no evidence to show whether this degree of fermentation would be adequate in limiting putrefaction, but the release of free amino acids indicates a reduction in complex proteins. Furthermore, the distinct reduction in protease-inhibiting ANFs shows potential in improving enzymatic digestion. The degradation of biogenic amines in some fermented products may enhance their safety and overall quality. Polyphenols and phytic acid from some fermentations may decrease negative effects from some putrefactive compounds; however, they may also impede protein digestion.

### 8.3 Mixed fermentations

Individual and combined fermentations of *Lb. helveticus* and *Saccharomyces cerevisiae* in pea protein showed an increase in ACE-inhibitory activity following *in vitro* gastrointestinal digestion compared to the non-fermented control. Intracellular proteases produced by *S. cerevisiae* were hypothesized to be important in the co-cultures to allow the further processing of component peptides by the lactobacilli (Vermeirssen et al., 2003). In an optimized mixed SSF fermentation on 50% moisture soybean meal with *Lactobacillus acidophilus, Lactobacillus delbrueckii, Ligilactobacillus salivarius*, and *Clostridium butyricum*, increased and faster degradation of protein was observed, compared to monocultures, or at different moisture contents (Su et al., 2018). A mixed-strain fermentation using *K. marxianus* with *Lpb. plantarum* or *P. pentosaceus* was performed in a kidney bean dough, reducing anti-nutritional factors. A gut microbiome animal model showed a decrease in pathogenic Escherichia and Shigella species, some of which were associated with putrefaction, after 12-day consumption of fermented bread (Chen et al., 2023).

By-products from alcohol production, distilled dried grains from corn and rice were used as substrate for fermentation of *B. subtilis* and *Lpb. plantarum*. Proteolytic degradation was seen in both fermentations, with surface structure disruption increasing the *in vitro* digestibility of the fermented grains (Wang et al., 2018).

Angiotensin I-converting enzyme (ACE) inhibitory peptides are currently being studied as a cure for hypertension. In a co-fermentation of *Lpb*. *plantarum* and *R. arrhizus* in wholegrain oats, increased ACE-inhibitory peptides and soluble proteins were seen (Wu et al., 2018). Fermentation shows potential in adding health-promoting metabolites, as well as improving digestibility. An increase in the overall solubility of the protein implies a decrease in hydrophobicity, meaning digestive enzyme target sites are being exposed and hence improve gastrointestinal digestibility.

There is some degree of validation from the literature that combined fermentations of multiple types of microbes lead to superior fermentation of substrates. Not all these studies contained comprehensive nutritional analyses of protein; hence, that must be a point of focus in the future. Studies containing co-cultures often did not sterilize the substrate, so contamination may contribute in part to the effects being seen.

## 9 Resistant starch to mitigate putrefaction

Despite the review’s focus on fermentation, studies emphasize resistant starch (RS) for gut health, as TIM-2 models show RS inclusion prevents putrefaction by boosting saccharolytic fermentation (linked to SCFAs) and commensals like *Phocaeicola vulgatus*. Branched pectins, fermented in the distal end of the colon where putrefaction occurs, prioritize this beneficial process, countering proteolytic toxicity (Toden et al., 2006).

There are several vitamins, minerals, and other metabolites that humans do not endogenously produce and hence require external sources for them. Certain commensal members of the gut microbiome, especially certain strains of the genus *Bifidobacterium,* have been demonstrated to convert dietary components into bioactive molecules including vitamins. Several *Bifidobacterium* strains produce B-group vitamins, e.g., folate at high levels (*Bifidobacterium bifidum*) or low levels (*Bifidobacterium breve*), confirmed by increased fecal levels of the vitamin in animal and human studies. Lactic acid bacteria found commensally can also contribute to folate production. Some strains from the species *Limosilactobacillus reuteri* found in fermented foods were shown to produce corrinoids like vitamin B_12_ which is not found in adequate quantities in a typical plant-based diet. There have been other commensal species that have been shown *in vitro* to produce other important compounds like vitamin K, nicotinic acid, and secondary bile acids. Fermented foods can be rich in SCFAs, BCFAs, and free metabolites that could potentially drive growth of saccharolytic bacteria.

Looking at mechanistic evidence of how RS is processed in health-promoting fermentation pathways can inform on what members of the gut microbiome are responsible, and what specific compounds cause positive health effects. While fermentation does not add resistant starch to foods, this could also potentially inform ways of carrying out pre-fermentation of plant-based foods to maximize their nutritional value (He et al., 2017; LeBlanc et al., 2013). By selecting strains that can add those positive compounds or targeting them, and further studying specialized fermentations can be prioritized.

## 10 Perspectives on future studies

There are two points of interest that must be addressed to clearly indicate whether fermentation would be useful as a tool to prevent putrefaction of plant protein. Firstly, the mechanisms of putrefaction of plant protein in humans must be understood, as well as whether putrefactive compounds are truly associated with deleterious health conditions. Secondly, the interactions between unfermented proteins or fermented proteins and putrefactive bacteria must be compared after gastrointestinal digestion.

To answer the first question, double-blind human intervention studies may prove valuable in seeing changes in the gut microbiome on consumption of a high-plant-protein diet versus a normal omnivore and high-animal-protein diet. While this would not be perfect, as gut microbiome changes may occur over a significantly longer period, any changes in putrefactive bacteria may allow initial conclusions to be drawn. This, paired with cell signaling and molecular experimentation of the effects of putrefaction compounds on human cell lines, can help provide more understanding. Further animal trials with transgenic pigs or mice may elucidate these potential systems in a live body.

To connect the effects of fermented plant protein with putrefaction, a protocol similar to the first proposed experiment could be followed. Fermented plant proteins that show a high degree of protein digestibility, both *in vivo* and *in vitro,* can be chosen and used in an intervention study like the one previously described. Depending on the presence or absence of putrefactive bacteria in the fecal microbiome of participants fed fermented samples versus non-fermented, inferences can be drawn on the effectiveness of fermentation as a mitigation tool. Furthermore, GI disease animal models can be utilized and addition of fermented foods in feed can be used to determine if any change in gut microbiome or disease markers has been observed. Traditional fermented foods must also be examined in more controlled environments, as points of references for future bio-processing methodologies. While microbial consortia may vary greatly in spontaneous fermentations, large sample sizes may help to identify trends in microbe type, protein modifications, metabolites, and effects on cells and gut microbiota. There is some evidence to suggest that prolonged fermentation times may result in reduced amino acid scores due to microbes metabolizing certain amino acids (Çabuk et al., 2018), so more work must also be done in optimizing fermentation conditions as well.

## 11 Conclusion

As discussed in this narrative review, putrefaction is a complex process that produces harmful metabolites that have been implicated in inflammatory and chronic diseases of the gut. The shift toward plant-based diets, driven by a multitude of concerns, has highlighted the need to address the challenges associated with plant protein digestibility and the link between this and putrefaction. Plant-based proteins, while potentially environmentally sustainable, high in fiber, and rich in essential nutrients, often suffer from lower bioavailability, structural complexity, and the presence of ANFs. For these proteins to be a completely sustainable alternative, they must be capable of sustaining human nutritional needs. Fermentation offers a potential solution; by degrading ANFs, modifying protein structures, and breaking down complex proteins, fermentation can improve the digestibility and nutritional quality of plant proteins, reducing their potential for harmful putrefaction. For example, fermentation of legume proteins by LAB and fungi has been shown to reduce ANFs, increase amino acid bioavailability, and improve protein digestibility *in vitro*. The topic of putrefaction as demonstrated is a complex one; it results in the release of metabolites in the gut, and some of those metabolites have been shown to cause potential harm in controlled laboratory or animal models. However, currently, the evidence linking poor digestion of protein to CRC, UC, and other gastrointestinal disorders is not strong enough to be conclusive. There is currently a lack of experimental evidence showing direct links between fermented food consumption and protein putrefaction mitigation in humans, or even a definitive case for putrefactive compounds consistently causing gastrointestinal disease. Most studies to date have been primarily conducted using *in vitro* digestibility or using *in vivo* animal models (rats, weaning pigs), limiting their direct applicability to humans. Furthermore, the variability in plant protein sources, processing methods, and microbial consortia used in fermentation necessitates more standardized and controlled human trials to validate these findings. The degree of fermentation, and ways to utilize it as a bio-processing tool must be identified, to reach a conclusion on optimal fermentation conditions.
